# Clinical and microbiological effects of *Lactobacillus reuteri* probiotics in the treatment of chronic periodontitis: a randomized placebo-controlled study

**DOI:** 10.1111/jcpe.12155

**Published:** 2013-09-15

**Authors:** Wim Teughels, Andaç Durukan, Onur Ozcelik, Martine Pauwels, Marc Quirynen, Mehmet Cenk Haytac

**Affiliations:** 1 Periodontology Section Department of Oral Sciences Faculty of Medicine, Catholic University LeuvenLeuven, Belgium; 2 Periodontology Section Dentistry, University Hospitals LeuvenLeuven, Belgium; 3 Fund for Scientific Research Flanders (FWO)Brussels, Belgium; 4 Department of Periodontology Faculty of Dentistry, Cukurova UniversityAdana, Turkey

**Keywords:** *Lactobacillus reuteri*, microbiology, periodontitis, probiotics, scaling and root planing, treatment

## Abstract

Teughels W, Durukan A, Ozcelik O, Pauwels M, Quirynen M, Haytac MC. Clinical and microbiological effects of Lactobacillus reuteri probiotics in the treatment of chronic periodontitis: a randomized placebo-controlled study. J Clin Periodontol 2013; 40: 1025–1035. doi: 10.1111/jcpe.12155.

**Aim**The aim of this randomized placebo-controlled clinical trial was to evaluate the effects of *Lactobacillus reuteri-*containing probiotic lozenges as an adjunct to scaling and root planing (SRP).

**Material and Methods**Thirty chronic periodontitis patients were recruited and monitored clinically and microbiologically at baseline, 3, 6, 9 and 12 weeks after therapy. All patients received one-stage full-mouth disinfection and randomly assigned over a test (SRP + probiotic, *n* = 15) or control (SRP + placebo, *n* = 15) group. The lozenges were used two times a day for 12 weeks.

**Results**At week 12, all clinical parameters were significantly reduced in both groups, while there was significantly more pocket depth reduction (*p* < 0.05) and attachment gain (*p* < 0.05) in moderate and deep pockets; more *Porphyromonas gingivalis* reduction was observed in the SRP + probiotic group.

**Conclusions**The results indicate that oral administration of *L. reuteri* lozenges could be a useful adjunct to SRP in chronic periodontitis.

It is well recognized that both the host and the bacterial challenge are key factors in the development of periodontal diseases. In the case of periodontitis, the periodontal destruction is substantially mediated by the host and driven by the bacterial challenge (Sanz & Quirynen 2005, Sanz & van Winkelhoff 2011). The presence of pathogenic bacteria, the absence of so-called “beneficial bacteria” and the susceptibility of the host are the main aetiological factors of periodontal diseases (Slots & Rams 1991, Socransky & Haffajee 1992, Wolff et al. 1994). Despite this knowledge, initial therapy focuses on the first factor, the reduction of periopathogens (Salvi & Lang 2005). It primarily encompasses scaling and root planing (SRP) and oral hygiene instructions (Haffajee et al. 2006). Although initially the number of pathogens can be greatly reduced by SRP, periodontopathogens quickly re-colonize the treated niches in the oral cavity (Teughels et al. 2007). This re-colonization process is left upon chance. The resulting shift to a less pathogenic composition of the subgingival microbiota is only temporary, even when combined with antiseptics or antibiotics (Quirynen et al. 2002). In this context, the administration of beneficial bacteria has emerged as a promising concept in the prevention and treatment of periodontal diseases. Since some beneficial bacteria posses both antimicrobial as well as anti-inflammatory properties, a whole new treatment approach might emerge where one aims to increase the proportion of beneficial bacteria in the oral cavity by either probiotics or prebiotics.

The World Health Organization describes probiotics as living microorganisms that confer health benefit on the host when administered in sufficient doses (http://www.who.int/entity/foodsafety/fs_management/en/probiotic_guidelines.pdf). Although this definition might not be 100% accurate anymore (Adams 2010), the potential benefits of probiotics on systemic health and medical disorders, such as gastrointestinal diseases, have been elaborately described (Broekaert & Walker 2006). In contrast, it is sparsely investigated what impact probiotics have on oral health (Meurman 2005) and in particular on periodontal diseases (Teughels et al. 2008, 2011). Teughels et al. (2007) described that the application of beneficial bacteria in beagle dogs after SRP resulted in a delayed and reduced re-colonization of periopathogens and reduced inflammation. In humans, reductions in bleeding upon probing, plaque index (PI) and gingivitis index (GI) after the application of probiotics have been reported (Krasse et al. 2005, Kang et al. 2006, Riccia et al. 2007, Twetman et al. 2009, Harini & Anegundi 2010). Also, microbiological effects have been observed (Sugano & Ito 2000, Ishikawa et al. 2003, Zahradnik et al. 2009). On the other hand, different studies have shown no clinical effects of probiotics (Matsuoka et al. 2006, Mayanagi et al. 2009, Iniesta et al. 2012, Hallstrom et al. 2013). However, almost all studies concerning probiotics and periodontal health are based on research in healthy or (experimental) gingivitis patients. In addition, most of these studies were carried out on patients with established biofilms, which are difficult therapeutic targets (Socransky & Haffajee 2002). Since antibiotics are more effective when the biofilm is disrupted and since, at least in vitro, for example, *Lactobacillus rhamnosus* GG can only establish itself in a biofilm when inoculated simultaneously with a salivary microcosm (Pham et al. 2009), it can be hypothesized that disruption of the biofilm is necessary prior to the administration of a probiotic. To date, only one study has been published involving the use of probiotics in periodontitis patients after SRP, which can be considered an act of biofilm disruption (Vivekananda et al. 2010). Although in this study the probiotics were administered 21 days after SRP, all clinical parameters were significantly better for SRP combined with the probiotics when compared to probiotics alone, SRP combined with a placebo or the placebo alone.

Therefore, the hypothesis of this study was that the adjunctive use of *Lactobacillus reuteri-*containing lozenges immediately after SRP in adult periodontitis patients would lead to improved clinical and microbiological outcomes when compared to SRP alone. The objective of this study was to evaluate the clinical and microbiological outcomes of the adjunctive use of this probiotic for 12 weeks after SRP in comparison to SRP combined with a placebo.

## Materials and Methods

This double-blind placebo-controlled parallel-arm clinical trial was approved by the local Ethical Committee of the Cukurova University, Adana, Turkey (CUDHF-EK 2009-3). Patients seeking for periodontal care or referred for periodontal care to the Department of Periodontology of the dental school of the Cukurova University were screened for the study. Inclusion criteria were: (1) healthy, non-institutionalized male or female patients, (2) at least 35 years of age, (3) a minimum of three natural teeth in each quadrant, excluding third molars, (4) previously untreated moderate to severe generalized adult periodontitis (Van der Velden 2005). Exclusion criteria were as follows: (1) having received antibiotics for any purpose within 6 months prior to entering the study or the need for antibiotic coverage for dental treatment (2) pregnancy and nursing, (3) acute oral lesions or necrotizing ulcerative periodontitis, (4) a history of diabetes, rheumatic fever, liver or kidney disease, neurological deficiencies, immunological diseases or use of medication which may affect periodontal tissue, (phenytoin, cyclosporin, nifidepine, chronic use of non-steroidal anti-inflammatory drugs), (5) current smoker or smoker over the past year.

Patients fulfilling the inclusion and exclusion criteria were invited to participate in the study. A written informed consent was obtained from all participants after a thorough explanation of the purpose, the nature, the implications and the potential risks and benefits of participating in this study. No changes in the trial design were made after approval by the local Ethics Committee.

### Sample size calculation

Sample size was calculated for the primary outcome variable, change in probing pocket depth (PPD), based on Vivekananda et al. (2010). Considering a standard deviation of 0.61 mm and a difference between the test and control group of 0.82 mm, it was calculated that 10 patients were needed in each group to provide 80% power with an *α* of 0.05 (version 2.7.3; StatsDirect, Cheshire, UK). Despite this low number and based on power calculations performed in studies comparing the effect of adjunctive antibiotics, it was decided to include 15 patients in each group.

### Experimental design and treatment protocol

Baseline examination consisted of full-mouth PPD, gingival recession (REC), bleeding on probing (BOP), measured at six sites per tooth. The full-mouth GI according to Löe & Silness (1963) and full-mouth PI was calculated according to Silness & Löe (1964). All examinations were performed using a North Carolina periodontal probe (Hu-Friedy, Chicago, IL, USA). After baseline examination, all patients received proper oral hygiene instructions and were given the same toothpaste (Colgate Total®; Colgate-Palmolive, Istanbul, Turkey) to be used during the entire study period. Initial periodontal therapy consisted of a full-mouth one-stage disinfection approach (Quirynen et al. 2006). Briefly, the patients were asked to rinse for 2 min. with a 0.12% chlorhexidine solution (Oroheks®; TriPharma, Istanbul, Turkey). SRP was performed on two consecutive days using an ultrasonic scaler (EMS, Nyon, Switzerland) under 0.12% chlorhexidine irrigation and using hand instruments. All mucosal surfaces were disinfected with CHX on a swap. All clinical manipulations were performed by one periodontist (AD).

The participants were randomized by the study coordinator (MCH) over the two treatment groups [control (SRP) or 12 week probiotic (SRP + P)]. The SRP group used a placebo lozenge two times a day for 12 weeks. The SRP + P group used a probiotic lozenge two times a day for 12 weeks.

At 3, 6, 9 weeks after initial treatment, follow-up visits were planned. At these appointments, microbial samples were taken and a clinical evaluation (PI, GI) was performed. At the final visit, 12 weeks after initial treatment, all baseline parameters were re-evaluated (PPD, REC, BOP, GI, PI, microbial analysis).

### Randomization

Randomization of the 30 patients, fulfilling the inclusion/exclusion criteria and willing to participate in this study, over the two different treatment groups was done by block randomization (version 2.7.3; StatsDirect). Coded bottles were given by the study coordinator (MCH) to the examiner (OO) at the patient's first initial treatment, 3, 6 and 9 week visit. Except for the study co-ordinator, all study personnel and patients were blinded to the study group assignment. Prior to sending the data to the biostatistician, the code was partially broken by MCH to group the different patients over the two treatments. Only after the statistical analysis, the designation of the different groups was revealed.

### Product under investigation

The probiotic lozenges consisted of *L. reuteri* (1 × 10^8^ CFU) for each of the strains DSM17938 and ATCC PTA5289 (Prodentis; BioGaia, Lund, Sweden). Both the probiotic and placebo lozenges could not be discriminated from each other by shape, texture or taste. The patients were asked to suck one lozenge in the morning and one at night, after tooth brushing and were instructed not to use any probiotic containing products during the course of the study.

### Microbiological analysis

#### Sample collection

At baseline, 3, 6, 9 and 12 weeks after therapy, saliva, supra- and subgingival samples were taken. Saliva samples were obtained by collecting 1 ml unstimulated saliva in a sterile vial. Pooled supragingival plaque samples were collected from 4 single rooted teeth, one in each quadrant showing the deepest PPD at baseline. Before sampling with Gracey curettes, the sites were isolated from saliva using cotton rolls and then gently dried with compressed air, to avoid contamination. All supragingival plaque from these sites was dispersed in 0.75 ml of TE (10 mM Tris-HCl, 1 mM EDTA, pH 7.6). An equal amount of 0.5 M NaOH was added to each Eppendorf tube. Samples were dispersed using a vortex mixer and immediately frozen at −20°C until analysis. Subgingival plaque samples were obtained from the same four teeth. Two paper-points (#35; Dentsply Maillefer, Ballaigues, Switzerland) were inserted (one mesial, one distal) until resistance was felt in each pocket of each tooth. After 10 s, the paper-points were transferred to a sterile Eppendorf tube, as described for supragingival plaque samples.

#### Microbiological processing

When the study was finished, the frozen samples were sent to the department of Periodontology of the KU Leuven (Belgium) on dry ice by express service and immediately frozen at −80°C upon arrival. After defrosting, 400 μl of each sample was centrifuged at 13,000 *g*. The obtained pellet was dispersed in 200 μl Instagene. DNA was extracted with InstaGene matrix (Bio-Rad Life Science Research, Hercules, CA, USA) according to the instructions of the manufacturer. Five micro litres of the purified DNA was used for the quantification of *Tannerella forsythia* (Shelburne et al. 2000), *Porphyromonas gingivalis* (Boutaga et al. 2003), *Aggregatibacter actinomycetemcomitans*, *Fusobacterium nucleatum* and *Prevotella intermedia* by qPCR as described previously (Boutaga et al. 2005). As a standard for the qPCR, a fragment of the 16S rRNA gene of *T. forsythia* ATCC 43037, *P. gingivalis* ATCC 33277, *A. actinomycetemcomitans* ATCC 43718, *F. nucleatum* ATCC 10953 and *P. intermedia* ATCC 25611 was amplified with primers flanking the annealing site of the qPCR primers. This fragment was ligated into the pGEM-T easy vector system (Promega, Madison, WI, USA). More details on the procedure can be found in Van Assche et al. (2009). Results were expressed as log 10 genome equivalents (gEq)/ml. All microbiological evaluations were performed blind.

### Outcome variables

Primary outcome variable was PPD. Secondary outcome variables were REC, BOP, percentage of sites showing gingival bleeding, percentage of sites showing plaque and microbiological variables. Clinical attachment level (CAL) was calculated as the sum of the PPD and REC.

Sub-analyses were performed on these outcome variables taking into account the initial PPD. A pocket was considered moderate if its initial PPD was between 4 and 6 mm and deep if ≥7 mm.

“Risk for disease progression” was defined at a patient level according to Lang & Tonetti (2003) as low (≤4 sites with PPD ≥5 mm), moderate (5–8 sites with PPD ≥5 mm) or high (≥9 sites with PPD ≥5 mm).

The “need for surgery” outcome measure was calculated according to Cionca et al. (2009). A site was considered as “in need for surgery” if the PPD was ≥6 mm or 5 mm and BOP positive. A tooth was considered in need for surgery if it had at least one site in need for surgery. A patient was considered in need for surgery if at least one tooth was in need for surgery.

### Examiner calibration

The clinical examiner (OO) was calibrated on 10 non-study periodontitis patients by measuring one quadrant in each subject with at least six teeth. The examiner measured PPD and CAL in the given quadrant and 60 min later, this same was measured again. The intra-examiner variability's for PPD and CAL measurements were assessed and determined to be 0.16 mm for PD and 0.19 mm for CAL.

### Compliance and adverse events

The patients returned the bottles containing the probiotic or placebo lozenges at the 3, 6, 9 and 12 week visit, to check for compliance. Each time, the patients received a new bottle with lozenges (MCH). Each time, the clinical examiner (OO) inquired the patient in relation to general health changes, use of anti-inflammatory drugs, use of mouth rinses, use of probiotic products and any adverse events that the patient might have noticed (e.g. gastrointestinal disturbances).

### Statistical analysis

Differences in continuous variables between SRP and SRP + P were established by means of a linear mixed model. Model assumptions were assessed by means of a normal quantile plot and residual dot plot.

Differences in binary variables were assessed by means of a generalized linear mixed model for binary outcomes with a logit-link. Patient and tooth, nested in patient, were taken as random variables. For linear mixed models for tooth-related parameters, only patient was used as a random factor.

Bacterial counts were taken into account by two different approaches: once as log10-transformed data, and once as a binary variable reflecting presence of absence of a certain species in a sample.

## Results

**Figure 1:**
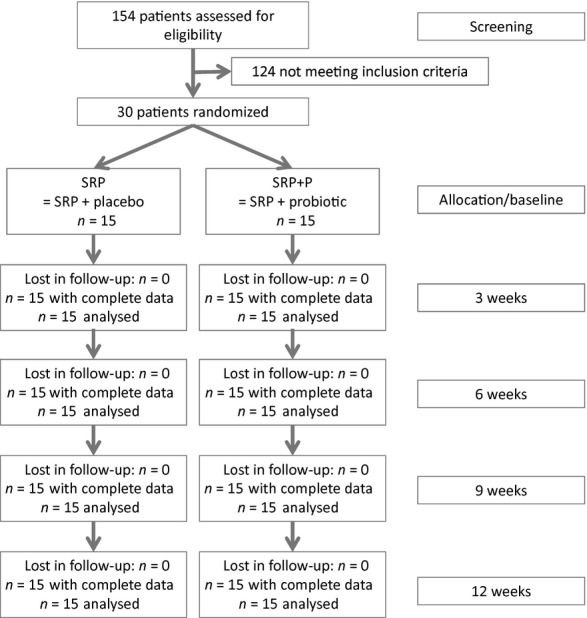
Flow chart

The flow chart of the study is shown in Fig. 1. The study patient demographics are shown in Table 1. No significant differences (*p* > 0.05) were shown between groups. All clinical data recording and clinical manipulations were performed between October 2009 and September 2010. All patients entering the study also completed the study. No compliance problems (as determined by counting the returned placebo or probiotic lozenges) were noted and no adverse effects of the product under investigation were mentioned by the patients or observed by the investigators.

**Table 1:** Demographic characteristics

Variable	Treatment group		*p-*value
	SRP Mean ± SD	SRP + P Mean ± SD	
Number of patients	15	15	NS
Number of males	8	7	NS
Number of smokers	0	0	NS
Age	45.73 ± 6.24	46.60 ± 4.47	NS

Significance of differences between groups: *p *> 0.1: not significant (NS); *p *< 0.1 to >0.05: tendency; *p *< 0.05: significant (bold).

SRP, scaling and rootplaning + placebo lozenge; SRP + P, scaling and rootplaning + probiotic lozenge.

### Probing pocket depth

As shown in Table 2, both patient groups were similar in mean PPD at baseline. Treatment resulted in significant reductions in full-mouth PPD (*p* < 0.05). No statistically significant inter-group differences were observed in the amount of full-mouth PPD reduction as well as in the full-mouth PPD at 12 weeks. However, the SRP + P group tended to have a lower full-mouth PPD at the end of the study when compared to the control group (*p* = 0.097). When analysing the data more in depth, by looking at pocket depth specific subgroups, there was a significant (*p* < 0.05) lower mean PPD at the end of the study in the SRP + P group for deep pockets when compared to the SRP group. Moderate pockets tended (*p* = 0.055) to be lower in the SRP + P group at 12 weeks when compared to SRP. For moderate and deep pockets, the SRP + P group showed significantly larger PPD reductions (*p* < 0.05) when compared to the SRP group.

**Table 2:** Mean (±standard deviation) probing pocket depth (PPD) outcome measures at baseline and 12 weeks

Variable	Time point	Treatment group				*p*-value	
		SRP		SRP + P		For mean	For delta
		Mean ± SD	Delta ± SD	Mean ± SD	Delta ± SD		
PPD (mm)							
Overall	Baseline	4.32 ± 0.50		4.15 ± 0.71		NS	
	12 weeks	2.93 ± 0.40*	−1.39 ± 0.15	2.73 ± 0.57*	−1.41 ± 0.25	0.097	NS
Moderate pockets	Baseline	4.84 ± 0.12		4.77 ± 0.24		NS	
	12 weeks	3.12 ± 0.22*	−1.72 ± 0.17	2.94 ± 0.40*	−1.84 ± 0.22	0.055	**0.041**
Deep pockets	Baseline	7.21 ± 0.25		7.27 ± 0.57		NS	
	12 weeks	4.95 ± 0.41*	−2.25 ± 0.27	4.39 ± 0.48*	−2.88 ± 0.35	**<0.001**	**<0.001**
% Sites with PPD							
≥5 mm	Baseline	44.85% ± 13.52		40.21% ± 19.81		NS	
	12 weeks	10.89% ± 7.40*		5.92% ± 11.83*		**0.004**	
≥6 mm	Baseline	19.84%* ± *11.79		15.88%* ± *15.03		NS	
	12 weeks	4.05%* ± *3.93*		2.89%* ± *6.32*		0.064	
≥7 mm	Baseline	10.69% ±7.48		9.69% ±12.71		NS	
	12 weeks	0.82%* ± *1.38*		0.51%* ± *1.61*		NS	
% Teeth with PPD							
≥5 mm	Baseline	87.31%* ± *11.31		81.16%* ± *19.07		NS	
	12 weeks	34.87%* ± *19.33*		17.08%* ± *23.74*		**0.004**	
≥6 mm	Baseline	54.71%* ± *23.99		44.58%* ± *26.76		NS	
	12 weeks	16.05%* ± *14.44*		11.06%* ± *19.53*		0.081	
≥7 mm	Baseline	36.18%* ± *21.35		29.50%* ± *24.75		NS	
	12 weeks	3.44%* ± *5.48*		2.22%* ± *6.46*		NS	
Number of patients with PPD							
≥5 mm	Baseline	15		15		NS	
	12 weeks	15		11		NS	
≥6 mm	Baseline	15		15		NS	
	12 weeks	13		9*		NS	
≥7 mm	Baseline	15		15		NS	
	12 weeks	6*		3*		NS	
Number of patients according to risk for disease progression (Lang & Tonetti 2003)							
Low		2/15 (13.3%)		9/15 (60.0%)		**0.027**	
Medium		3/15 (20.0%)		2/15 (13.3%)			
High		10/15 (66.6%)		4/15 (26.6%)			

Significance of differences between groups: *p *> 0.1: not significant (NS); *p *< 0.1 to > 0.05: tendency; *p *< 0.05: significant (bold).

* Significantly different from baseline.

SRP, scaling and rootplaning + placebo lozenge; SRP + P, scaling and rootplaning + probiotic lozenge.

When the data were analysed in terms of percentage of sites or percentage of teeth with a PPD ≥5, ≥6 or ≥7 mm, no significant differences (*p* > 0.05) were detected at baseline. Both treatments resulted in significant reductions (*p* < 0.05) in these parameters. At 12 weeks, the SRP + P group showed a significantly lower percentage of sites and percentage of teeth with a PPD ≥5 mm (*p* < 0.05). For percentage of sites and percentage of teeth with a PPD ≥6 mm, SPR + P tended to have lower values when compared to SRP (*p* = 0.064 and 0.081). In terms of percentage of sites or percentage of teeth with PPD ≥7 mm and in terms of number of patients with a PPD ≥5, ≥6 or ≥7 mm, no significant differences (*p* > 0.05) or tendencies (*p* > 0.1) between both groups could be shown although the SRP + P group consistently resulted in better outcomes when compared to SRP.

Analysing the SRP and SRP + P data according to the individual risk profile for periodontal disease progression (Lang & Tonetti 2003) showed that significantly fewer patients had a high risk for disease progression and significantly more patients had a low risk for disease progression when they received the SRP + P treatment (*p* < 0.027).

### CAL, REC, BOP

As shown in Table 3, no significant inter-group differences were observed at baseline and at 12 weeks for CAL, REC and BOP (*p* > 0.05). However, the CAL gain tended (*p* = 0.074) to be more pronounced in the SRP + P group. More specifically, both in initially moderate and deep pockets, there was a significantly (*p* < 0.05) greater gain in CAL for the SRP + P group when compared to the SRP group. In addition, the pockets in the SRP + P group tended (*p* = 0.089) to show less recession formation over the 12 week period.

**Table 3:** Mean (±standard deviation) CAL, REC, BOP and need for surgery outcome measures at baseline and 12 weeks

Variable	Time point	Treatment group				*p*-value	
		SRP		SRP + P			
		Mean ± SD	Delta ± SD	Mean ± SD	Delta ± SD	For mean	For delta
CAL (mm)							
Overall	Baseline	4.97 ± 0.61		4.97* ± *1.01		NS	
	12 weeks	4.21* ± *0.67*	−0.76* ± *0.36	3.97* ± *0.97	−0.99* ± *0.22	NS	0.074
Moderate pockets	Baseline	5.49* ± *0.31		5.60* ± *0.60*		NS	
	12 weeks	4.48* ± *0.55*	−1.01* ± *0.59	4.18* ± *0.70	−1.42* ± *0.27	NS	**0.014**
Deep pockets	Baseline	7.77* ± *0.43		8.19* ± *1.34		NS	
	12 weeks	7.10* ± *0.78*	−0.68* ± *0.85	6.72* ± *1.06*	−1.47* ± *0.71	NS	**0.007**
REC (mm)							
Overall	Baseline	0.66* ± *0.73		0.82* ± *0.71		NS	
	12 weeks	1.28* ± *0.42*	0.63* ± *0.31	1.24 ± 0.75*	0.42 ± 0.18	NS	0.089
BOP (%)							
Overall	Baseline	67.53%* ± *11.37		70.70%* ± *14.53		NS	
	12 weeks	16.58%* ± *10.54*		15.51%* ± *11.92*		NS	
% Sites in need for surgery							
Overall	Baseline	41.17%* ± *12.2		38.42%* ± *30.33		NS	
	12 weeks	6.44%* ± *5.45*		5.03%* ± *9.92*		0.081	
Moderate pockets	Baseline	59.80%* ± *5.77		60.88%* ± *20.17		NS	
	12 weeks	3.81%* ± *3.31*		2.96%* ± *4.76*		NS	
Deep pockets	Baseline	100%* ± *0		100%* ± *0		NS	
	12 weeks	34.43%* ± *21.30*		18.30%* ± *23.00*		**0.045**	
% Teeth in need for surgery							
Overall	Baseline	82.27%* ± *14.36		78.58%* ± *20.02		NS	
	12 weeks	25.17%* ± *18.38*		15.74%* ± *23.34*		0.067	
Number of patients in need for surgery							
0 sites		2/15 (13.3%)		5/15 (33.3%)		**0.019**	
1-2 sites		0/15 (0%)		4/15 (26.6%)			
≥ 3 sites		13/15 (86.6%)		6/15 (40.0%)			

Significance of differences between groups: *p *> 0.1: not significant (NS); *p *< 0.1 to > 0.05: tendency; *p *< 0.05: significant (bold).

* Significantly different from baseline.

SRP, scaling and rootplaning + placebo lozenge; SRP + P, scaling and rootplaning + probiotic lozenge; REC, gingival recession, BOP, bleeding on probing.

Based on the PPD and BOP data, a “need for surgery” outcome measure was calculated according to Cionca et al. (2009). As shown in Table 3, the patients in the SRP + P groups tended (*p* = 0.081) to show less sites and less teeth in need for surgery at 12 weeks when compared to the SRP group. Moreover, initially deep sites showed a significantly (*p* < 0.05) lower need for surgery at 12 weeks when they received the SRP + P treatment instead of SRP alone. In addition, significantly (*p* < 0.05) fewer patients were classified as needing surgery on ≥3 teeth when they received the SRP + P treatment.

### Plaque and gingival bleeding

Table 4 summarizes the percentage of sites showing plaque or gingival bleeding over the time course of the clinical trial. Although both outcome measures were consistently lower in the SRP + P group, only on a few occasions, these differences were statistically significant between both treatment groups.

**Table 4:** Mean (±standard deviation) percentage of plaque and gingival bleeding outcome measures at baseline and 12 weeks

Variable	Time point	Treatment group		*p*-value
		SRP Mean ± SD	SRP + P Mean ± SD	For mean
% Sites with supragingival plaque				
Overall	Baseline	99.66* ± *0.99	95* ± *10.27	NS
	3 weeks	25.25* ± *31.27*	8.94* ± *12.20*	0.089
	6 weeks	26.18 ± 31.42*	10.91 ± 16.59*	**0.029**
	9 weeks	25.77 ± 31.33*	11.13 ± 13.28*	NS
	12 weeks	24.88 ± 33.26*	16.34 ± 19.19*	NS
% Sites with gingival bleeding				
Overall	Baseline	99.57 ± 1.16	97.77 ± 4.38	NS
	3 weeks	32.9 ± 30.2*	13.54 ± 16.59*	0.074
	6 weeks	33.45 ± 30.01*	16.37 ± 19.91*	0.089
	9 weeks	30.89 ± 29.54*	14.06 ± 16.51*	NS
	12 weeks	29.01 ± 32.36*	4.3 ± 10.69*	**<0.001**

Significance of differences between groups: *p *> 0.1: not significant (NS); *p *< 0.1 to >0.05: tendency; *p *< 0.05: significant (bold).

* Significantly different from baseline.

SRP, scaling and rootplaning + placebo lozenge; SRP + P, scaling and rootplaning + probiotic lozenge.

### Microbiology

The microbiological data for the supra-, subgingival and saliva samples are shown in Tables 5, 6 and 7. As shown, significantly (*p* < 0.05) larger reductions in *P. gingivalis* numbers were found in the subgingival, supragingival and saliva samples in the SRP + P group over the 12 week period, when compared to the SRP group. There was also a tendency detected for less *P. gingivalis* in the saliva at 9 and 12 weeks in the SRP + P group (*p* = 0.085 and 0.098). In addition, *P. intermedia* tended to show a larger reduction and smaller numbers in the supragingival plaque samples at week 12 of the SRP + P group (*p* = 0.074 and 0.085). In saliva, *P. intermedia* numbers in the SRP + P group were significantly (*p* < 0.05) lower at week 12 when compared to the SRP group. For all other outcome measures, including detection frequencies (data not shown), no significant microbiological differences could be found between both treatment groups.

**Table 5:** Mean (±standard deviation) for microbiological outcome measures in subgingival plaque

Species	Time point	Mean log10 cfu/ml* ± *SD			Δ mean log10 cfu/ml		
		SRP	SRP + P	*p*-value	SRP	SRP + P	*p*-value
*Aggregatibacter actinomycetemcomitans*	Baseline	3.57* ± *1.97	3.84 ± 2.7	NS			
	3 weeks	0.69 ± 1.83*	0.63 ± 1.76*	NS	−3.32 ± 1.03	−4.38 ± 1.78	NS
	6 weeks	0.77 ± 2.05*	0.97 ± 2.15*	NS	−3.22 ± 1.18	−3.92 ± 2.08	NS
	9 weeks	0.96 ± 2.12*	1.19 ± 2.38*	NS	−3.01 ± 1.25	−3.61 ± 2.19	NS
	12 weeks	1.86 ± 2.12*	1.98 ± 2.38*	NS	−1.98 ± 1.23	−2.53 ± 1.98	NS
*Fusobacterium nucleatum*	Baseline	7.4 ± 1.16	7.7 ± 1.11	NS			
	3 weeks	2.94 ± 2.25*	2.95 ± 1.82*	NS	−4.46 ± 2.15	−4.75 ± 2.37	NS
	6 weeks	3.71 ± 2.09*	4.38 ± 2.22*	NS	−3,69 ± 2.07	−3.32 ± 2.39	NS
	9 weeks	4.92 ± 1.39*	4.6 ± 2.03*	NS	−2.47 ± 1.61	−3.1 ± 2.22	NS
	12 weeks	5.87 ± 1.08*	5.45 ± 2.05*	NS	−1.53 ± 1.31	−2.25 ± 1.89	NS
*Porphyromonas gingivalis*	Baseline	6.37 ± 1.7	6.67 ± 1.5	NS			
	3 weeks	3.26 ± 1.47*	3.17 ± 1.71*	NS	−3.11 ± 1.54	−3.5 ± 1.48	NS
	6 weeks	4.08 ± 1.61*	3.89 ± 1.23*	NS	−2.3 ± 1.37	−2.79 ± 1.31	NS
	9 weeks	4.98 ± 1.5*	4.23 ± 1.23*	NS	−1.39 ± 0.79	−2.44 ± 1.43	**0.034**
	12 weeks	5.43 ± 1.73*	4.87 ± 1.21*	NS	−0.94 ± 0.61	−1.8 ± 1.17	**0.050**
*Prevotella intermedia*	Baseline	6.17 ± 2.73	6.34 ± 2.14	NS			
	3 weeks	2.47 ± 1.7*	2.22 ± 2.02*	NS	−4,27 ± 1.79	−4.12 ± 1.88	NS
	6 weeks	3.55 ± 1.75*	2.7 ± 2.06*	NS	−3.02 ± 1.21	−3.63 ± 1.85	NS
	9 weeks	4.04 ± 1.96*	3.35 ± 1.97*	NS	−2.45 ± 1.45	−2.99 ± 1.7	NS
	12 weeks	4.81 ± 2.44*	4.22 ± 2.07*	NS	−1.57 ± 1.21	−2.12 ± 1.7	NS
*Tannerella forsythia*	Baseline	6.56 ± 0.89	5.95 ± 1.82	NS			
	3 weeks	1.34 ± 2*	1.71 ± 2.18*	NS	−5.22 ± 1.72	−4.57 ± 1.93	NS
	6 weeks	2.96 ± 2.38*	3.56 ± 2.13*	NS	−3.6 ± 2.15	−2.56 ± 1.45	NS
	9 weeks	4.22 ± 1.99*	4.02 ± 2.12*	NS	−2.34 ± 1.81	−2.07 ± 1.39	NS
	12 weeks	5.24 ± 1.17*	4.96 ± 1.78*	NS	−1.33 ± 0.94	−1.06 ± 0.81	NS
Total load	Baseline	10.11 ± 0.86	9.9 ± 0.52	NS			
	3 weeks	7.34 ± 1.22*	6.93 ± 1.54*	NS	−2.78 ± 0.92	−2.97 ± 1.66	NS
	6 weeks	7.76 ± 1.01*	8.04 ± 1.06*	NS	−2.36 ± 0.77	−1.86 ± 1.03	NS
	9 weeks	8.42 ± 1.19*	8.24 ± 0.98*	NS	−1.69 ± 1.07	−1.66 ± 1.02	NS
	12 weeks	8.99 ± 0.99*	8.49 ± 0.82*	NS	−1.12 ± 1.04	−1.41 ± 0.7	NS

Significance of differences between groups: *p *> 0.1: not significant (NS); *p *< 0.1 to >0.05: tendency; *p *< 0.05: significant (bold).

* Significantly different from baseline.

SRP, scaling and rootplaning + placebo lozenge; SRP + P, scaling and rootplaning + probiotic lozenge.

**Table 6:** Mean (±standard deviation) for microbiological outcome measures in supragingival plaque

Species	Time point	Mean log10 cfu/ml ± SD			Δ mean log10 cfu/ml		
		SRP	SRP + P	*p*-value	SRP	SRP +P	*p*-value
*Aggregatibacter actinomycetemcomitans*	Baseline	3.43 ± 2.29	3.58 ± 3.32	NS			
	3 weeks	0.62 ± 1.63*	0.79 ± 2.13*	NS	−3.52 ± 1.27	−4.64 ± 2.19	NS
	6 weeks	0.63 ± 1.7*	1.4 ± 2.56*	NS	−3.49 ± 1.36	−3.62 ± 2.01	NS
	9 weeks	1.04 ± 1.94*	1.65 ± 2.85*	NS	−2.99 ± 1.2	−3.21 ± 2.19	NS
	12 weeks	1.21 ± 2.08*	2.18 ± 2.83*	NS	−2.78 ± 1.4	−2.32 ± 1.48	NS
*Fusobacterium nucleatum*	Baseline	7.12 ± 1.32	6.45 ± 1.66	NS			
	3 weeks	1.7 ± 2.49*	1.14 ± 2.02*	NS	−5.42 ± 2.21	−5.3 ± 1.6	NS
	6 weeks	3.11 ± 2.69*	2.99 ± 2.47*	NS	−4.01 ± 2.41	−3.45 ± 1.42	NS
	9 weeks	4.17 ± 2.62*	3.82 ± 2.39*	NS	−2.94 ± 2.31	−2.63 ± 1.19	NS
	12 weeks	5.47 ± 1.71*	4.48 ± 2.32*	NS	−1.65 ± 1.2	−1.96 ± 1.19	NS
*Porphyromonas gingivalis*	Baseline	6.93 ± 1.61	7.17 ± 1.48	NS			
	3 weeks	3.97 ± 1.68*	3.49 ± 1.58*	NS	−2.97 ± 1.68	−3.68 ± 1.2	0.089
	6 weeks	4.72 ± 1.79*	4.62 ± 1.22*	NS	−2.21 ± 1.48	−2.55 ± 1	NS
	9 weeks	5.53 ± 1.9*	4.9 ± 1.31*	NS	−1.4 ± 0.82	−2.27 ± 0.84	**0.009**
	12 weeks	6.05 ± 2.04*	5.06 ± 1.5*	NS	−0.89 ± 0.67	−2.11 ± 0.84	**0.000**
*Prevotella intermedia*	Baseline	7.64 ± 1.56	6.77 ± 2.45	NS			
	3 weeks	3.04 ± 2.24*	2.79 ± 2.19*	NS	−4.6 ± 2.03	−4.26 ± 1.65	NS
	6 weeks	4.32 ± 2.16*	3.28 ± 2.28*	NS	−3.32 ± 1.7	−3.73 ± 1.69	NS
	9 weeks	4.93 ± 2.41*	4.22 ± 1.91*	NS	−2.71 ± 1.93	−2.73 ± 1	NS
	12 weeks	6.08 ± 1.54*	4.85 ± 1.86*	0.074	−1.56 ± 1.15	−2.05 ± 0.81	0.085
*Tannerella forsythia*	Baseline	7.25 ± 0.68	6.89 ± 0.56	NS			
	3 weeks	1.49 ± 2.18*	2.28 ± 2.38*	NS	−5.76 ± 2.01	−4.61 ± 2.17	NS
	6 weeks	3.58 ± 2.34*	4.23 ± 1.94*	NS	−3.67 ± 2.41	−2.67 ± 1.56	NS
	9 weeks	4.68* ± *2*	4.61* ± *1.99*	NS	−2.57* ± *1.91	−2.28* ± *1.67	NS
	12 weeks	5.74* ± *1.25*	5.42* ± *1.27*	NS	−1.51* ± *1.23	−1.48* ± *1.07	NS
Total load	Baseline	10.56* ± *0.74	10.92* ± *0.72	NS			
	3 weeks	8* ± *1.91*	8.8* ± *1.27*	NS	−2.55* ± *1.75	−2.12* ± *1.21	NS
	6 weeks	9.15* ± *1.09*	9.07* ± *1.25*	NS	−1.41* ± *0.92	−1.85* ± *1.04	NS
	9 weeks	9.51* ± *1.11*	9.67* ± *1.16*	NS	−1.04* ± *0.98	−1.26* ± *1.01	NS
	12 weeks	9.89* ± *0.84*	9.96* ± *0.97*	NS	−0.66* ± *0.62	−0.97* ± *0.89	NS

Significance of differences between groups; *p *> 0.1: not significant (NS); *p *< 0.1 to > 0.05: tendency; *p *< 0.05: significant (bold).

* Significantly different from baseline.

SRP, scaling and rootplaning + placebo lozenge; SRP + P, scaling and rootplaning + probiotic lozenge.

**Table 7:** Mean (±standard deviation) for microbiological outcome measures in saliva

Species	Time point	Mean log10 cfu/ml* ± *SD			Δ mean log10 cfu/ml		
		SRP	SRP + P	*p*-value	SRP	SRP + P	*p*-value
*Aggregatibacter actinomycetemcomitans*	Baseline	3.07* ± *2.06	3.02* ± *2.86	NS			
	3 weeks	0.68* ± *1.8*	0.66* ± *1.82*	NS	−2.99* ± *0.78	−3.93* ± *1.91	NS
	6 weeks	0.75* ± *2*	1.07* ± *2.27*	NS	−2.9* ± *0.95	−3.26* ± *1.95	NS
	9 weeks	1.07* ± *2.3*	1.17* ± *2.46*	NS	−2.51* ± *1.24	−3.09* ± *2.11	NS
	12 weeks	1.31* ± *2.45*	1.67* ± *2.66*	NS	−2.2* ± *1.54	−2.25* ± *1.6	NS
*Fusobacterium nucleatum*	Baseline	6.8* ± *1.03	5.86* ± *2.17	NS			
	3 weeks	1.34* ± *2.46*	0.51* ± *1.29*	NS	−5.46* ± *2.11	−5.35* ± *2.2	NS
	6 weeks	3.2* ± *2.71*	3.2* ± *2.6*	NS	−3.6* ± *2.44	−2.65* ± *1.64	NS
	9 weeks	4.32* ± *2.6*	3.64* ± *2.75*	NS	−2.48* ± *2.29	−2.22* ± *1.66	NS
	12 weeks	5.82* ± *1.34*	4.83* ± *2.47*	NS	−0.98* ± *0.83	−1.02* ± *1.19	NS
*Porphyromonas gingivalis*	Baseline	6.7* ± *1.72	6.92* ± *1.33	NS			
	3 weeks	3.9* ± *2.02*	2.94* ± *2.11*	NS	−2.8* ± *2.21	−3.99* ± *2.28	0.093
	6 weeks	4.88* ± *1.77*	4.42* ± *1.51*	NS	−1.82* ± *1.64	−2.5* ± *1.31	0.071
	9 weeks	5.73* ± *1.98*	5.23* ± *1.01*	0.085	−0.96* ± *0.85	−1.69* ± *0.94	**0.011**
	12 weeks	6.48* ± *1.61*	5.75* ± *0.96*	0.098	−0.22* ± *0.21	−1.17* ± *0.76	**0.000**
*Prevotella intermedia*	Baseline	7.57* ± *1.13	6.74* ± *1.49	NS			
	3 weeks	2.06* ± *2.41*	2.09* ± *2.28*	NS	−5.51* ± *2.4	−4.65* ± *2.22	NS
	6 weeks	4.73* ± *1.76*	3.6* ± *2.14*	NS	−2.84* ± *2.04	−3.14* ± *1.71	NS
	9 weeks	5.59* ± *1.34*	4.06* ± *2.38*	0.085	−1.98* ± *1.61	−2.68* ± *1.89	NS
	12 weeks	6.7* ± *1.27*	5.37* ± *1.34*	**0.017**	−0.87* ± *0.92	−1.37* ± *0.89	NS
*Tannerella forsythia*	Baseline	6.7* ± *0.55	6* ± *1.84	NS			
	3 weeks	1.26* ± *1.99*	1.75* ± *2.27*	NS	−5.44* ± *1.97	−4.56* ± *2.07	NS
	6 weeks	3.5* ± *2.47*	3.98* ± *2.11*	NS	−3.2* ± *2.4	−2.17* ± *1.27	NS
	9 weeks	4.92* ± *2.06*	4.59* ± *2.25*	NS	−1.78* ± *1.79	−1.52* ± *1.35	NS
	12 weeks	6.11* ± *1.08*	5.36* ± *1.82*	NS	−0.59* ± *0.73	−0.69* ± *0.67	NS
Total load	Baseline	10.82* ± *0.5	10.3* ± *0.35	NS			
	3 weeks	7.76* ± *1.38*	7.5* ± *1.37*	NS	−3.06* ± *1.35	−2.8* ± *1.47	NS
	6 weeks	9.18* ± *0.97*	8.7* ± *1.02*	NS	−1.64* ± *0.9	−1.6* ± *1.01	NS
	9 weeks	9.76* ± *0.93*	9.21* ± *0.91*	NS	−1.06* ± *0.85	−1.1* ± *0.87	NS
	12 weeks	10.31* ± *0.77*	9.74* ± *0.65*	0.074	−0.51* ± *0.55	−0.56* ± *0.5	NS

Significance of differences between groups: *p *> 0.1: not significant (NS); *p *< 0.1 to > 0.05: tendency; *p *< 0.05: significant (bold).

* Significantly different from baseline.

SRP, scaling and rootplaning + placebo lozenge; SRP + P, scaling and rootplaning + probiotic lozenge.

## Discussion

This double-blinded placebo-controlled RCT evaluated the effect of the adjunctive use of *L. reuteri-*containing lozenges after SRP, two times a day for 3 months, on clinical and microbiological parameters in chronic periodontitis patients. It was shown that there was a benefit for the patients using the *L. reuteri* lozenges. In relation to the primary outcome variable, significantly larger PPD reductions, especially in deep pockets, and significantly lower percentages of sites and teeth with a residual pocket depth of ≥5 mm were evident. This resulted in significantly more patients falling in the low category in terms of risk for disease progression according to Lang & Tonetti (2003). In addition, patients using the probiotic lozenges gained significantly more attachment in moderate and deep pockets. At the end of the study, these patients had significantly less deep pockets that were classified as in need for surgery and significantly less patients were classified as needing surgery on ≥3 teeth. Also, significantly more pronounced reductions in *P. gingivalis* numbers were observed.

To the best of our knowledge, the underlying study is the first study that reports on the clinical and microbiological effects of probiotic supplementation as an adjunct to SRP in the treatment of chronic periodontitis. In an attempt to improve the impact of the probiotic lozenges, the probiotic application was started immediately after a full-mouth disinfection procedure (Teughels et al. 2011). There is, however, one study with a similar set-up and size, using the same probiotic lozenges at a similar concentration and frequency recently published (Vivekananda et al. 2010). However, in the latter study, the patients started to use the probiotic lozenges 21 days after SRP and no additional disinfection of the oral cavity was performed. In addition, the follow-up time was considerably shorter (21 days). Comparing the results of both studies, with these differences in mind, it is clear that the results of our study are clearly inferior to those of Vivekananda et al. (2010) who reported significant inter-group differences in PI, GI, gingival bleeding index (%), PPD, CAL, and the number of *A. actinomycetemcomitans*, *P. gingivalis* and *P. intermedia* in favour of the use of *L. reuteri* probiotic lozenges. Of these, the only difference which could be confirmed at a level of significance was the lower number *P. gingivalis* species when *L. reuteri* probiotics were used. Also, Iniesta et al. (2012) reported this effect. This can be of significance since *P. gingivalis* is considered as a keystone pathogen which can create a dysbiosis between the host and dental plaque (Darveau et al. 2012). One should however acknowledge that the patients in the Vivekananda study were more severely diseased since the average PPD was 5.17 mm. This could partially explain the different results between both studies since, as shown in our study for PPD and CAL, the deeper the pocket at baseline, the more pronounced the effect of the probiotic was. Other potential factors which could hypothetically explain our more inferior results are the time between SRP and the start of the probiotic application, the use of chlorhexidine during SRP to further suppress the microbiological ecology and the time between the start of the probiotic lozenges and the moment of evaluation. Regarding the latter aspect, it should be noted that in terms of, for example, average PPD reduction, both studies report a similar PPD reduction for the SRP + P group (1.31 ± 0.49 mm *versus* 1.41 ± 0.25 mm). However, there is a difference in the mean PPD reduction for the control groups between both studies (0.49 ± 0.39 mm *versus* 1.39 ± 0.15 mm). Taking into account the different follow-up times (42 days *versus* 12 weeks), this might indicate that the use of the probiotic lozenges results in a faster PPD reduction initially.

The most striking result of the study was the observation that at the end of the study 66.7% (*n* = 10) of the patients in the control group and only 26.7% (*n* = 4) of the patients in the SRP + P group fell into the high risk for disease progression category proposed by Lang & Tonetti (2003). These percentages are comparable, if not identical, to what is reported in different studies using amoxicillin combined with metronidazole as an adjunct to SRP (Feres et al. 2012, Mestnik et al. 2012). Also, the lower percentage of patients, teeth and sites classified as in need for surgery according to (Cionca et al. 2009) at the end of the study was enlightening although the authors stress that this decision should always be made at the level of the individual patient. Nevertheless, these two more unconventional outcome measures emphasize the clinical benefit and significance of the use of these *L. reuteri* lozenges under the given conditions.

The major limitation of this study was its power. Obviously since only one similar study existed (Vivekananda et al. 2010), there was not a lot of data to perform a power analysis. Although a power calculation was performed a priori (*n* = 10/group) and the authors even increased the number of patients (*n *= 15/group), still a lot of the outcome measures (e.g. difference in PPD, percentage of sites and teeth with PPD ≥6 mm, change in CAL and REC, percentage of sites and teeth in need for surgery) only tended to be different between both groups. Since the main problem of low powered RCT's is the increased probability of type II error (false negative), the study might have been too small to detect actual differences between groups (Mestnik et al. 2012). Therefore, the authors also put emphasis on observed tendencies when analysing the data. A post hoc power calculation, based on the primary outcome measure for this study, indicates that 63 subjects are needed per group to provide 80% power with an *α* of 0.05. Despite this, still a lot of significant and clinically relevant differences were observed in favour of the use of the probiotic lozenges.

Another limitation of the study could be that the colonization of *L. reuteri* was not evaluated. The reasons for this were technical. During the course of the study, no specific (quantitative) PCR procedure was available that specifically could detect *L. reuteri* DSM17938 or ATCC PTA5289 without cross-amplification of other *Lactobacillus* species (Jacobsen et al. 1999) or which could differentiate between *L. reuteri* strains in multi-species samples (Dommels et al. 2009). A culturing technique (Caglar et al. 2009) and a technique which combines culturing with a colony PCR (Romani et al. 2013) has been described but since the samples were frozen and analysed in Belgium, this technique could not be applied. Since colonization and even viability are not specific requirements for probiotics to exert beneficial effects, for example, in the gastrointestinal tract (Teughels et al. 2008, 2011, Adams 2010), no attempt was made to detect or quantify the *L. reuteri* strains. Moreover, such analysis would not have made a change in the conclusions of this study.

In conclusion, this study showed that under the given conditions the adjunctive use of *L. reuteri* lozenges resulted in significant additional clinical improvements primarily for initially moderate to deep pockets when compared to SRP alone. The microbiological differences were more moderate and primarily restricted to *P. gingivalis* numbers. This questions the conclusion of a recent literature review which states that the effects of probiotic bacteria on periodontal clinical parameters are much more restricted than on the microbiological results (Teughels et al. 2011). However, the latter conclusion was based on studies that did not use probiotics as an adjunct to SRP. Despite this, the clinical results showed a clinically relevant benefit for the patient as “risk for disease progression” and “need for additional surgery” outcome measures were significantly better when *L. reuteri* lozenges were used as an adjunct to SRP. It needs to be emphasized that these results cannot be generalized to other probiotic products or modes of application (Teughels et al. 2011).

**Clinical Relevance**

*Scientific rationale for the study*: The effects probiotics as an adjunct to scaling and root planing in non-surgical periodontal therapy of chronic periodontitis patients are hardly known.

*Principal findings*: Under the given conditions, the probiotic therapy resulted in additional clinical benefits in moderate and deep pockets and in lower *P. gingivalis* numbers.

*Practical implications*: The use of this probiotic supplement during 3 months as an adjunct to scaling and root planing can be considered a valuable treatment option.

## References

[b1] Adams CA (2010). The probiotic paradox: live and dead cells are biological response modifiers. Nutritional Research Reviews.

[b2] Boutaga K, van Winkelhoff AJ, Vandenbroucke-Grauls CM, Savelkoul PH (2003). Comparison of real-time PCR and culture for detection of *Porphyromonas gingivalis* in subgingival plaque samples. Journal of Clinical Microbiology.

[b3] Boutaga K, van Winkelhoff AJ, Vandenbroucke-Grauls CM, Savelkoul PH (2005). Periodontal pathogens: a quantitative comparison of anaerobic culture and real-time PCR. FEMS Immunology and Medical Microbiology.

[b4] Broekaert IJ, Walker WA (2006). Probiotics and chronic disease. Journal of Clinical Gastroenterology.

[b5] Caglar E, Topcuoglu N, Cildir SK, Sandalli N, Kulekci G (2009). Oral colonization by *Lactobacillus reuteri* ATCC 55730 after exposure to probiotics. International Journal of Paediatric Dentistry.

[b6] Cionca N, Giannopoulou C, Ugolotti G, Mombelli A (2009). Amoxicillin and metronidazole as an adjunct to full-mouth scaling and root planing of chronic periodontitis. Journal of Periodontology.

[b7] Darveau RP, Hajishengallis G, Curtis MA (2012). *Porphyromonas gingivalis* as a potential community activist for disease. Journal of Dental Research.

[b8] Dommels YE, Kemperman RA, Zebregs YE, Draaisma RB, Jol A, Wolvers DA, Vaughan EE, Albers R (2009). Survival of *Lactobacillus reuteri* DSM 17938 and *Lactobacillus rhamnosus* GG in the human gastrointestinal tract with daily consumption of a low-fat probiotic spread. Applied Environmental Microbiology.

[b9] Feres M, Soares GM, Mendes JA, Silva MP, Faveri M, Teles R, Socransky SS, Figueiredo LC (2012). Metronidazole alone or with amoxicillin as adjuncts to non-surgical treatment of chronic periodontitis: a 1-year double-blinded, placebo-controlled, randomized clinical trial. Journal of Clinical Periodontology.

[b10] Haffajee AD, Teles RP, Socransky SS (2006). The effect of periodontal therapy on the composition of the subgingival microbiota. Periodontology 2000.

[b11] Hallstrom H, Lindgren S, Yucel-Lindberg T, Dahlen G, Renvert S, Twetman S (2013). Effect of probiotic lozenges on inflammatory reactions and oral biofilm during experimental gingivitis. Acta Odontologica Scandinavia.

[b12] Harini PM, Anegundi RT (2010). Efficacy of a probiotic and chlorhexidine mouth rinses: a short-term clinical study. Journal of the Indian Society of Pedodontics and Preventive Dentistry.

[b13] Iniesta M, Herrera D, Montero E, Zurbriggen M, Matos AR, Marin MJ, Sanchez-Beltran MC, Llama-Palacio A, Sanz M (2012). Probiotic effects of orally administered *Lactobacillus reuteri*-containing tablets on the subgingival and salivary microbiota in patients with gingivitis. A randomized clinical trial. Journal of Clinical Periodontology.

[b14] Ishikawa H, Aiba Y, Nakanishi M, Oh-hashi Y, Koga Y (2003). Suppression of periodontal pathogenic bacteria in the saliva of humans by the administration of *Lactobacillus salivarius* TI 2711. Journal of the Japanese Society of Periodontology.

[b15] Jacobsen CN, Rosenfeldt NV, Hayford AE, Moller PL, Michaelsen KF, Paerregaard A, Sandstrom B, Tvede M, Jakobsen M (1999). Screening of probiotic activities of forty-seven strains of Lactobacillus spp. by in vitro techniques and evaluation of the colonization ability of five selected strains in humans. Applied Environmental Microbiology.

[b16] Kang MS, Chung J, Kim SM, Yang KH, Oh JS (2006). Effect of *Weissella cibaria* isolates on the formation of *Streptococcus mutans* biofilm. Caries Research.

[b17] Krasse P, Carlsson B, Dahl C, Paulsson A, Nilsson A, Sinkiewicz G (2005). Decreased gum bleeding and reduced gingivitis by the probiotic *Lactobacillus reuteri*. Swedish Dental Journal.

[b18] Lang NP, Tonetti MS (2003). Periodontal risk assessment (PRA) for patients in supportive periodontal therapy (SPT). Oral Health and Preventive Dentistry.

[b19] Löe H, Silness J (1963). Periodontal disease in pregnancy I. Prevalence and severity. Acta Odontologica Scandinavia.

[b20] Matsuoka T, Sugano N, Takigawa S, Takane M, Yoshinuma N, Ito K, Koga Y (2006). Effect of oral *Lactobacillus salivarius* TI 2711 administration on periodontopathic bacteria in subgingival plaque. Journal of the Japanese Society of Periodontology.

[b21] Mayanagi G, Kimura M, Nakaya S, Hirata H, Sakamoto M, Benno Y, Shimauchi H (2009). Probiotic effects of orally administered Lactobacillus salivarius WB21-containing tablets on periodontopathic bacteria: a double-blinded, placebo-controlled, randomized clinical trial. Journal of Clinical Periodontology.

[b22] Mestnik MJ, Feres M, Figueiredo LC, Soares G, Teles RP, Fermiano D, Duarte PM, Faveri M (2012). The effects of adjunctive metronidazole plus amoxicillin in the treatment of generalized aggressive periodontitis: a 1-year double-blinded, placebo-controlled, randomized clinical trial. Journal of Clinical Periodontology.

[b23] Meurman JH (2005). Probiotics: do they have a role in oral medicine and dentistry?. European Journal of Oral Sciences.

[b24] Pham LC, van Spanning RJ, Roling WF, Prosperi AC, Terefework Z, Ten Cate JM, Crielaard W, Zaura E (2009). Effects of probiotic *Lactobacillus salivarius* W24 on the compositional stability of oral microbial communities. Archives of Oral Biology.

[b25] Quirynen M, De Soete M, Boschmans G, Pauwels M, Coucke W, Teughels W, van Steenberghe D (2006). Benefit of “one-stage full-mouth disinfection” is explained by disinfection and root planing within 24 hours: a randomized controlled trial. Journal of Clinical Periodontology.

[b26] Quirynen M, Teughels W, De Soete M, van Steenberghe D (2002). Topical antiseptics and antibiotics in the initial therapy of chronic adult periodontitis: microbiological aspects. Periodontology 2000.

[b27] Riccia DN, Bizzini F, Perilli MG, Polimeni A, Trinchieri V, Amicosante G, Cifone MG (2007). Anti-inflammatory effects of *Lactobacillus brevis* (CD2) on periodontal disease. Oral Diseases.

[b28] Romani VN, Hasslof P, Keller MK, Granstrom E, Roos S, Twetman S, Stecksen-Blicks C (2013). *Lactobacillus reuteri* influences regrowth of Mutans streptococci after Full-Mouth Disinfection: a double-blind, randomised controlled trial. Caries Research.

[b29] Salvi GE, Lang NP (2005). The effects of non-steroidal anti-inflammatory drugs (selective and non-selective) on the treatment of periodontal diseases. Current Pharmaceutical Design.

[b30] Sanz M, Quirynen M (2005). Advances in the aetiology of periodontitis. Group A consensus report of the 5th European Workshop in Periodontology. Journal of Clinical Periodontology.

[b31] Sanz M, van Winkelhoff AJ (2011). Periodontal infections: understanding the complexity–consensus of the Seventh European Workshop on Periodontology. Journal of Clinical Periodontology.

[b32] Shelburne CE, Prabhu A, Gleason RM, Mullally BH, Coulter WA (2000). Quantitation of *Bacteroides forsythus* in subgingival plaque comparison of immunoassay and quantitative polymerase chain reaction. Journal of Microbiological Methods.

[b33] Silness J, Löe H (1964). Periodontal disease in pregnancy. II. Correlation between oral hygiene and periodontal condition. Acta Odontologica Scandinavia.

[b34] Slots J, Rams TE (1991). New views on periodontal microbiota in special patient categories. Journal of Clinical Periodontology.

[b35] Socransky SS, Haffajee AD (1992). The bacterial etiology of destructive periodontal disease: current concepts. Journal of Periodontology.

[b36] Socransky SS, Haffajee AD (2002). Dental biofilms: difficult therapeutic targets. Periodontology 2000.

[b37] Sugano N, Ito K (2000). Nicotine switches the form of H(2)O(2)-induced cell death from apoptosis to necrosis in U937 cells. Immunology Letters.

[b38] Teughels W, Loozen G, Quirynen M (2011). Do probiotics offer opportunities to manipulate the periodontal oral microbiota?. Journal of Clinical Periodontology.

[b39] Teughels W, Newman MG, Coucke W, Haffajee AD, van der Mei HC, Haake SK, Schepers E, Cassiman JJ, Van Eldere J, van Steenberghe D, Quirynen M (2007). Guiding periodontal pocket recolonization: a proof of concept. Journal of Dental Research.

[b40] Teughels W, Van Essche M, Sliepen I, Quirynen M (2008). Probiotics and oral healthcare. Periodontology 2000.

[b41] Twetman S, Derawi B, Keller M, Ekstrand K, Yucel-Lindberg T, Stecksen-Blicks C (2009). Short-term effect of chewing gums containing probiotic *Lactobacillus reuteri* on the levels of inflammatory mediators in gingival crevicular fluid. Acta Odontologica Scandinavia.

[b42] Van Assche N, Van Essche M, Pauwels M, Teughels W, Quirynen M (2009). Do periodontopathogens disappear after full-mouth tooth extraction?. Journal of Clinical Periodontology.

[b43] Van der Velden U (2005). Purpose and problems of periodontal disease classification. Periodontology 2000.

[b44] Vivekananda MR, Vandana KL, Bhat KG (2010). Effect of the probiotic *Lactobacilli reuteri* (Prodentis) in the management of periodontal disease: a preliminary randomized clinical trial. Journal of Oral Microbiology.

[b45] Wolff L, Dahlen G, Aeppli D (1994). Bacteria as risk markers for periodontitis. Journal of Periodontology.

[b46] Zahradnik RT, Magnusson I, Walker C, McDonell E, Hillman CH, Hillman JD (2009). Preliminary assessment of safety and effectiveness in humans of ProBiora(3), a probiotic mouthwash. Journal of Applied Microbiology.

